# The Many Functions of Foxp3^+^ Regulatory T Cells in the Intestine

**DOI:** 10.3389/fimmu.2020.600973

**Published:** 2020-10-20

**Authors:** Catalina Cosovanu, Christian Neumann

**Affiliations:** Institute of Microbiology, Infectious Diseases and Immunology, Charité-Universitätsmedizin, Berlin, Germany

**Keywords:** Treg cell, functions, phenotypes, intestinal tolerance, microbiota, diet, tissue homeostasis, intestinal epithelial cells

## Abstract

Throughout the last years, gut-resident Foxp3^+^ regulatory T (Treg) cells have been associated with a growing number of tissue-specific functions in the intestine, comprising various aspects of gut immunity and physiology. Treg cells have pivotal roles in intestinal tolerance induction and host defense by actively controlling immune responses towards harmless dietary antigens and commensal microorganisms as well as towards invading pathogens. In addition to these immune-related roles, it has become increasingly clear that intestinal Treg cells also exert important non-immune functions in the gut, such as promoting local tissue repair and preserving the integrity of the epithelial barrier. Thereby, intestinal Treg cells critically contribute to the maintenance of tissue homeostasis. In order to account for this functional diversity, gut-resident Treg cells have specifically adapted to the intestinal tissue microenvironment. In this Review, we discuss the specialization of Treg cells in the intestine. We survey the different populations of gut-resident Treg cells focussing on their unique functions, phenotypes and distinct transcription factor dependencies.

## Introduction

One of the major functions of Foxp3^+^ Treg cells residing in non-lymphoid tissues is to control local inflammation. Given the overwhelming load of microbial and food antigens in the intestine, a cardinal function of gut-resident Foxp3^+^ Treg cells is to contain inflammatory immune responses to the microbiota and dietary factors, thereby establishing and maintaining intestinal immune tolerance. This essential role of gut-resident Foxp3^+^ Treg cells is highlighted by the development of immunodysregulation polyendocrinopathy enteropathy X-linked (IPEX) syndrome in patients who lack functional Foxp3^+^ Treg cells (1, 2). IPEX syndrome results in spontaneous inflammation of many organs, yet IPEX patients most frequently suffer from severe gastrointestinal disorders and food allergies (3, 4), emphasizing the key role of Foxp3^+^ Treg cells in establishing tolerance within the intestine. In addition to maintaining tolerance towards environmental antigens, gut-resident Foxp3^+^ Treg cells also shape immunity against invading intestinal pathogens by either suppressing or promoting inflammatory anti-pathogen immune responses, thus determining host susceptibility to intestinal infections. Furthermore, there is growing evidence that intestinal Foxp3^+^ Treg cells regulate many non-immunological processes in the gut. Indeed, important roles of Treg cells in e.g. local tissue repair and promotion of epithelial barrier functions are now emerging. These non-traditional roles have a profound impact on gut homeostasis and physiology and should therefore be considered as important facets of gut-resident Foxp3^+^ Treg cell function.

In accordance with this remarkable functional heterogeneity, gut-resident Foxp3^+^ Treg cells have acquired unique phenotypes, governed by specific transcriptional networks, that are tailored to the diverse challenges of the intestinal tissue microenvironment. In fact, the existence of functionally distinct Treg cell subsets in the gut, enabling a certain division of labour, can be considered as one of the key factors underlying intestinal homeostasis. In this regard, two developmental origins have been described for intestinal Foxp3^+^ Treg cells (5). The first occurs in the thymus, where thymus-derived Treg (tTreg) cells are generated following recognition of self-antigen by the T cell receptor. The second pathway of Treg cell generation is in peripheral tissues, such as the gut, where, under certain conditions, naïve CD4^+^ T cells develop into peripherally-derived Treg (pTreg) cells upon recognition of their cognate antigen, which is regarded as being non-self. Thus, intestinal pTreg cells are thought to be mainly responsible for tolerance to non-self-antigens, such as environmental antigens, whereas tTreg cells would be preferentially involved in controlling autoreactive responses. Phenotypically, expression of the markers Helios and Neuropilin 1 (Nrp1) by tTreg cells but not by pTreg cells can be used to distinguish these subsets (6–8), although this distinction is known to have exceptions (9–11).

In summary, in this Review, we will discuss the current understanding of Foxp3^+^ Treg cell adaptation in the intestine, including their specific functions, phenotypes and distinct transcription factor dependencies.

## Treg Cells Mediate Tolerance to Environmental Antigens

### Control of T Cell Responses to Microbial Antigens

**Figure 1:**
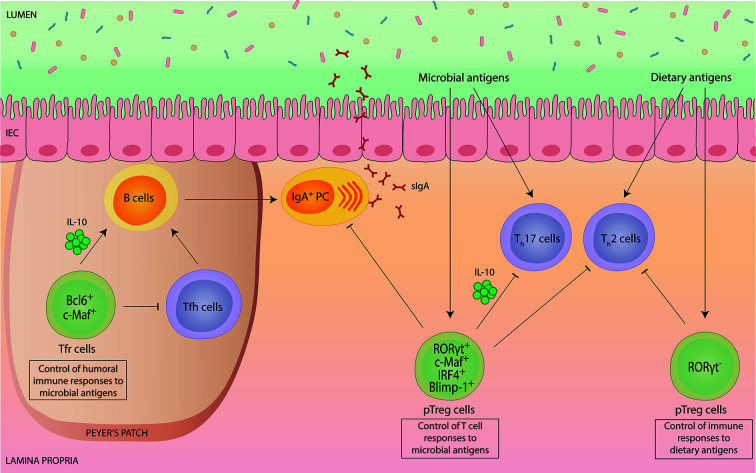
Intestinal Foxp3^+^ Treg cells mediate tolerance to environmental antigens.Intestinal Foxp3^+^ Treg cells are potent suppressors of immune responses to environmental antigens, such as commensal microbes and harmless dietary antigens. Peripherally-induced Helios^-^ Nrp1^-^ Foxp3^+^ Treg (pTreg) cells co-expressing RORγt differentiate specifically in response to microbial antigens and have a crucial role in the suppression of microbiota-specific Th17 cell and IgA responses. In addition, RORγt^+^ pTreg cells also contribute to the suppression of aberrant intestinal Th2 cell responses. Molecularly, RORγt^+^ pTreg cell differentiation and function, such as production of immunosuppressive IL-10, depends on the transcriptional regulators c-Maf, IRF4 and Blimp-1. pTreg cells induced by dietary antigens lack RORγt expression. This Treg cell subset has a specific role in installing tolerance to ingested antigens by controlling food-specific Th cell responses. Within Peyer’s Patches, specialized Foxp3^+^ T follicular regulatory (Tfr) cells depend on Bcl6 and c-Maf and exhibit a dual role in controlling the germinal center reaction and subsequent IgA production. While Tfr cells exert a suppressive effect on T follicular helper (Tfh) cell expansion and function, they can also promote B cell-mediated IgA secretion *via* IL-10.

Since their initial discovery, Foxp3^+^ Treg cells were recognized as potent suppressors of T cell responses. Accordingly, gut-resident Treg cells play a pivotal role in suppressing effector T cell responses to the microbiota (**Figure 1**). A subpopulation of Foxp3^+^ Treg cells found primarily in the large intestine, characterized by co-expression of the RAR-related orphan receptor γt (RORγt), has been suggested to specifically mediate tolerance to the microbiota (12). Indeed, induction and maintenance of RORγt^+^ Treg cells critically depend on the microbiota (13–15) and/or specific metabolites thereof, such as microbial secondary bile acids (16–18) or short chain fatty acids (SCFA) (15, 19–21), although the specific role of SCFA for RORγt^+^ Treg cells is controversial (14). In addition to microbial metabolites, food-derived vitamin A seems to specifically drive RORγt^+^ Treg cells in the intestine (15). RORγt^+^ Treg cells comprise the majority of the Helios^-^ Nrp1^-^ Foxp3^+^ pTreg cells in the intestine that differentiate locally in response to commensal microbes in an antigen-specific manner (22–25). Consistently, RORγt^+^ pTreg cells are selectively decreased in germ-free and antibiotic-treated mice (14, 15). Likewise, during postnatal development, the generation of RORγt^+^ pTreg cells coincides with the increased uptake of luminal antigens and diversification of the microbiota during weaning, which is critical for the development of tolerance to gut bacteria (26, 27).

Functionally, RORγt^+^ pTreg cells express particularly high levels of IL-10, CTLA-4 and ICOS, indicative of a superior suppressive capacity (13, 28). Especially, secretion of the anti-inflammatory cytokine IL-10 by Treg cells has proven to be essential for maintaining intestinal tolerance, as evidenced by the development of spontaneous colitis upon genetic deletion of IL-10 selectively in Foxp3^+^ cells (29). RORγt^+^ pTreg cells were shown to control intestinal inflammation in different models of colitis (13–15), although the specific role of RORγt^+^ pTreg cells has remained unclear, with different studies reporting different conclusions. Whereas one study proposed that RORγt^+^ pTreg cells are crucial in controlling aberrant Th2 cell responses (15), a finding that is consistent with the selective Th2 cell dysregulation observed in mice that specifically lack pTreg cells (30), another report observed selective control of Th1 and Th17 cells (14). This suggests that the function of RORγt^+^ pTreg cells is highly context-dependent and most likely influenced by the indigenous microbiota.

Our own work as well as that of others demonstrated a specific role of gut-resident Foxp3^+^ Treg cells in controlling intestinal microbiota-specific Th17 cell responses (31–34). Importantly, we identified the transcription factor c-Maf to be essential for gut-resident Treg cells to differentiate into RORγt^+^ pTreg cells, to express IL-10 and to maintain intestinal tolerance (31–34). Notably, in comparison to RORγt, c-Maf appears to have a more substantial role for the control of microbiota-specific T cell responses, as inflammatory Th17 cell accumulation and spontaneous intestinal inflammation was only observed upon Treg cell-specific deletion of c-Maf but not of RORγt (31, 32). Consistent with this, c-Maf-deficiency in Treg cells also resulted in gut dysbiosis and breakdown of host-microbiota homeostasis (32).

In accordance with the fact that expression of c-Maf (and RORγt) in Treg cells is dependent on STAT3 activation (15, 32, 35), uncontrolled intestinal Th17 cell responses and spontaneous colitis were also detected in Treg cell-specific STAT3-deficient mice (36). In addition to c-Maf, RORγt^+^ pTreg cells also co-express high levels of the transcription factor Blimp-1 (37). Blimp-1, together with IRF4, critically contributes to the control of IL-10 production in Treg cells (38, 39), although Foxp3^+^ Treg cell-specific deletion of Blimp-1 was not sufficient to cause severe chronic intestinal inflammation as it was observed in CD4^+^ T cell-specific Blimp-1-deficient mice (40).

Importantly, although tolerance induction to microbial antigens has been mainly attributed to pTreg cells, there is evidence that also naturally occurring tTreg cells contribute to this process (41).

### Control of Humoral Immune Responses to Microbial Antigens

In addition to the control of microbiota-specific T cell responses, gut-resident Foxp3^+^ Treg cells also play an important role in regulating humoral immune responses to the microbiota, such as intestinal immunoglobulin A (IgA) production and selection (**Figure 1**). IgA is the most abundant antibody in mucosal secretions and essential to intestinal homeostasis by both maintaining non-invasive commensal bacteria and neutralizing invasive pathogens (42). Early reports demonstrated a supportive role of Treg cells for intestinal IgA production based on the findings that depletion of Treg cells resulted in a rapid loss of intestinal IgA (43), and that Treg cells can contribute to the germinal center (GC) reaction in Peyer’s Patches (PPs) by conversion into T follicular helper (Tfh) cells (44). Later, a specialized subset of Foxp3^+^ Treg cells within follicles, termed T follicular regulatory (Tfr) cells, was identified (45–47). Tfr cells share many characteristics with Tfh cells, including the expression of PD-1, CXCR5, and dependency on the transcription factor Bcl6, which allows them to gain access to GCs while maintaining their suppressive capacity (45–47). Thus, Tfr cells can specifically suppress excessive Tfh cell-mediated B cell responses. Consistent with this, lack of Tfr cells was shown to result in dysregulated Tfh cells and IgA selection in PPs, thereby precipitating intestinal microbial dysbiosis (48).

Besides the suppressive effect of Tfr cells on GC, there is growing evidence that Tfr cells can also act as “helper” cells for humoral immune responses (49). Mechanistically, this positive effect of Tfr cells on GC is associated with Tfr cell-derived IL-10 production (50). Indeed, IL-10 is known to promote the proliferation of activated B cells and subsequent IgA production (51, 52), as well as the development and maintenance of intestinal microbiota-dependent IgA^+^ plasma cells (53). However, the relative contribution of Treg cell-derived IL-10 production for intestinal IgA production has remained unclear. We and others recently showed that intestinal Foxp3^+^ Treg cells require the transcription factor c-Maf to produce IL-10 and to adopt a Tfr cell phenotype (32, 33). Interestingly, Treg cell-specific deletion of c-Maf resulted in strongly elevated frequencies of lamina propria IgA^+^ plasma cells (32). While c-Maf clearly controls multiple Treg cell functions beyond their ability to produce IL-10, we also observed a slight increase in intestinal IgA levels in Treg cell-specific IL-10-deficient mice (32).

A very recent report discovered that microbiota-dependent RORγt^+^ pTreg cells and IgA^+^ B cells can regulate each other in a double-negative feedback loop that is transmitted through multiple generations (54). While these findings suggest that intestinal IgA level are also critically controlled by Foxp3^+^ Treg cells outside of follicles, the cellular and molecular entities involved in this reciprocal regulation remain to be defined. Notably, given that RORγt^+^ pTreg cell differentiation is dependent on c-Maf, these results also suggest that the hyper IgA phenotype of Treg cell-specific c-Maf-deficient mice is at least partially driven by the lack of direct suppression of RORγt^+^ pTreg cells on IgA (32). Collectively, these findings suggest a highly context-dependent function of Foxp3^+^ Treg cells for intestinal IgA regulation. Clearly, more work is needed to precisely define the role of Treg cells in regulating humoral immunity in the gut.

### Control of Immune Responses to Dietary Antigens

Aside from microbial antigens, dietary antigens represent a major source of natural antigenic stimulation in the gut. Tolerance to food antigens is characterized by the absence and/or suppression of antigen-specific immune responses, a phenomenon known as oral tolerance. Foxp3^+^ Treg cells play a central role in installing oral tolerance, as evidenced by the fact that loss-of-function mutations affecting Foxp3 in mice and humans result in spontaneous severe allergic inflammation, such as food allergies (FA) (4, 55). Likewise, inducible depletion or functional impairment of Foxp3^+^ Treg cells in mice tolerant to ovalbumin was shown to be sufficient to abolish oral tolerance, demonstrating a dominant role of antigen-specific Treg cells in conferring tolerance to ingested antigens (56, 57).

Among the intestinal Foxp3^+^ Treg cell populations, pTreg cells but not tTreg cells, appear to be essential for oral tolerance induction (5, 30, 58) (**Figure 1**). More specifically, analysis of germ-free mice fed with an elemental diet devoid of dietary antigens identified a specific pTreg cell population that was unaffected by the absence of the gut microbiota but disappeared upon antigen-free diet (59). These food-induced pTreg cells were distinguishable from microbiota-induced pTreg cells by their lack of RORγt expression (59). Importantly, without this population, mice showed an increased susceptibility to FA (59). Notably, although directed against the microbiota, ablation of RORγt^+^ pTreg cells also rendered mice more susceptible to FA (60). Vice versa, FA patients manifest dynamic microbial dysbiosis and RORγt^+^ pTreg cell-inducing microbiota therapy in mice promoted restoration of oral tolerance in FA (60), demonstrating a hitherto unrecognized mechanistical link between Treg cell-mediated tolerance induction to microbial and dietary antigens.

Other examples of how nutritional signals impact on mucosal immune responses stem from studies focussing on the manipulation of the host nutritional status (61–63). Intermittent fasting, for instance, was shown to strongly affect the abundance and functionality of intestinal lymphocytes, including Treg cells, as well as the susceptibility to inflammatory diseases (61, 62), highlighting the close link between diet, Treg cells and intestinal immune homeostasis.

## Treg Cells Control Intestinal Inflammation and Host Defense

### Control of Intestinal Inflammation and Tissue Damage

Gut-resident Foxp3^+^ Treg cells not only operate during homeostasis to establish and maintain a tolerogenic environment in the intestine. In fact, Treg cells are able to specifically sense inflammatory signals, which leads to their activation and heightening of their suppressive capacity to counteract e.g. inflammation and inflammation-driven tissue damage (64, 65).

**Figure 2:**
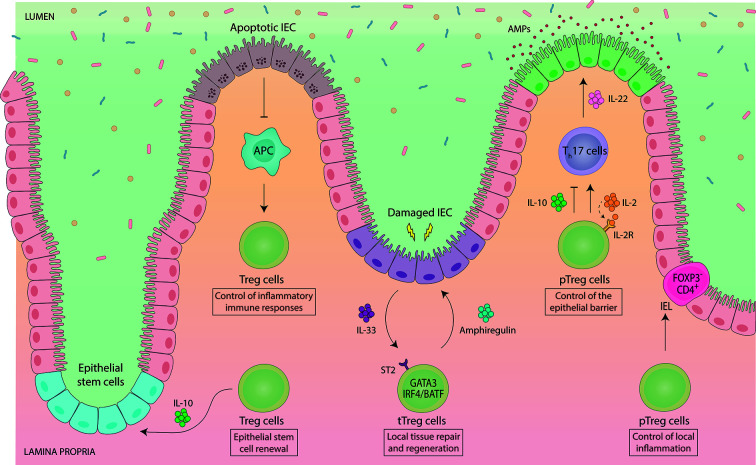
Intestinal Foxp3^+^ Treg cells engage in a functional crosstalk with intestinal epithelial cells. Gut-resident Foxp3^+^ Treg cells support tissue physiology by maintaining intestinal epithelial cell (IEC) homeostasis. Vice versa, IEC-derived signals control the abundance and functionality of intestinal Treg cells. Treg cell-derived IL-10 promotes the renewal of intestinal epithelial stem cells, while apoptotic IEC negatively regulate the proliferation and abundance of intestinal Treg cells. Thymically-induced Helios^+^ Nrp1^+^ Foxp3^+^ Treg (tTreg) cells co-expressing GATA3 have been implicated in local tissue repair and regeneration. GATA3^+^ tTreg cells express ST2, by which they can sense IL-33, an alarmin, which is produced by IEC e.g. upon infection-induced damage. In response, ST2^+^ GATA3^+^ tTreg cells get activated, expand and produce the growth factor amphiregulin. Developmentally, ST2^+^ GATA3^+^ tTreg cells rely on IRF4 and BATF for their differentiation. pTreg cells indirectly contribute to the maintenance of the epithelial barrier by controlling the abundance of IL-22-producing Th17 cells. IL-22 directly acts on IEC to control IEC growth, permeability, production of mucus and antimicrobial proteins (AMPs). While Treg cells are mostly presented as suppressors of Th17 cells, they can also promote Th17 cell responses *via* consumption of IL-2 during mucosal infections. Intestinal pTreg cells also show intra-tissue specialization. Upon migration to the IEC barrier, pTreg cells downregulate Foxp3 and become CD4^+^ Foxp3^-^ intraepithelial (IEL) T cells in order to control local inflammation, demonstrating a dominant role of the IEC microenvironment in controlling Treg cell lineage stability and plasticity.

A substantial fraction of intestinal Foxp3^+^ Treg cells has a phenotypic signature specifically linked to tissue repair, such as expression of ST2, the receptor for the alarmin IL-33, and the growth factor amphiregulin (66) (**Figure 2**). In addition, enhanced production and activation of IL-10 and TGF-ß has been detected in ST2^+^ Treg cells, demonstrating their highly activated and suppressive phenotype (67). ST2^+^ Treg cells co-express the canonical transcription factor of type 2 immunity GATA3, are mostly Helios^+^/Nrp1^+^ and are unaffected by the absence of the gut microbiota, indicative of a thymic origin (15). GATA3 directly interacts with Foxp3 both on protein and gene level to regulate expression of Foxp3 itself as well as the downstream Foxp3-dependent transcriptional program (68, 69). Developmentally, ST2^+^ tTreg cells rely on IRF4 and BATF for their differentiation (70–72).

Since IL-33 is primarily produced by intestinal epithelial cells upon local damage (73), and ST2^+^ tTreg cells exhibit high expression of the gut-homing receptors CCR9 and α4β7 (67, 74), the prevailing model for ST2^+^ tTreg cell function is that they specifically home to sites of damage in the intestine and mediate repair, although this has not been formally proven yet. In support of a specific role of ST2^+^ GATA3^+^ tTreg cells in controlling local inflammation, it was shown that GATA3 expression was not required at steady state, but was essential under inflammatory conditions to enable Treg cell accumulation at inflammatory sites (75). Furthermore, Treg cell-specific GATA3 deletion led to spontaneous inflammation in mice, including development of intestinal pathologies (68, 69), although these disorders were not present in young mice, but only observed upon aging (ca. after 6 months) (68, 69, 75, 76). Notably, since Helios^+^ Nrp1^+^ tTreg cells are thought to be positively selected against self-antigens, they may also be involved in preventing autoimmune inflammation in the gut. In an experimental system, in which a model self-antigen was specifically expressed in the intestinal epithelium, activation and expansion of autoreactive T cells was inhibited by self-antigen-specific tTreg cells (77).

While ST2^+^ GATA3^+^ tTreg cells are clearly linked to the regulation of type 2 inflammation, type 1 inflammatory immune responses appear to be specifically controlled by Foxp3^+^ Treg cells co-expressing the transcription factor T-bet (78, 79). In fact, type 1 inflammation selectively induces T-bet expression in Treg cells *via* IFN-γ- or IL-27-dependent signalling to endow Treg cells with the homeostatic and migratory properties required for the suppression of type 1 immune responses (76, 78–80). Functionally, T-bet^+^ Treg cells were shown to limit Th1-mediated autoimmune- or infection-induced pathology in the intestine but also at extra-intestinal sites (76, 78–80). However, T-bet^+^ Foxp3^+^ Treg cells may also acquire pro-inflammatory IFN-γ co-expression during intestinal inflammation, thereby promoting gut inflammatory diseases (81, 82).

### Control of Host Defense Against Intestinal Pathogens

Foxp3^+^ Treg cells are also important in regulating host defense against invading intestinal pathogens. In this context, it has become clear that the functional role of Treg cell-mediated control of immune responses to infectious agents is highly context-dependent, ranging from detrimental to advantageous outcomes for the host.

For instance, during intestinal helminth infection, Foxp3^+^ Treg cells are actively induced by the pathogen leading to a state of hyporesponsiveness, which is key for parasite persistence (83, 84). However, expansion of Treg cells not only enhances parasite survival but also protects the host from excessive type 2 inflammatory immune responses against the pathogen, thereby minimizing `collateral damage` to the gut tissue (85, 86). Notably, upon helminth infection, selective expansion of Foxp3^+^ Helios^+^ tTreg as well as Foxp3^+^ Helios^-^ pTreg cells has been described (87), suggesting that control of anti-helminth immunity involves multiple pathways of Treg cell recruitment. Indeed, ST2^+^ tTreg cells, activated and expanded by helminth-induced epithelial damage-mediated release of IL-33 (88), as well as pTreg cells, induced by helminth-derived secretory products (89), were shown to contribute to the control of mucosal helminth infection.

Importantly, in addition to suppressing anti-pathogen immunity, Foxp3^+^ Treg cells can also directly promote host-protective immune responses. Upon mucosal infections with *Citrobacter rodentium* or *Candida albicans*, Treg cells were shown to support protective Th17 cell responses by consumption of IL-2 (90–92), a potent inhibitor of Th17 cell differentiation (93). This supportive role stands in opposition to the suppressive function of Treg cells for microbiota-specific Th17 cell responses during homeostasis (see section above) (31–34, 36). Nevertheless, recent data indicate that Treg cells also participate in the inhibition of inflammatory pathogen-specific Th17 cell responses. For instance, susceptibility to infection with the intestinal protozoan parasite *Giardia lamblia* correlated with increased RORγt^+^ pTreg to Th17 cell ratios, suggesting that RORγt^+^ pTreg cells also contribute to the suppression of Th17 cells during intestinal infection, thereby hampering protective immunity (94). Similarly, induction of RORγt^+^ pTreg cells in response to the pathobiont *Helicobacter hepaticus* prevented expansion of pathogenic antigen-specific Th17 cells, thus enabling immunological tolerance (31).

## Treg Cells Preserve Gut Physiology

### Control of Epithelial Barrier Functions

A novel concept in immunology is that tissue-resident immune cells not only mediate immune homeostasis and host defense but also critically contribute to the maintenance of organismal physiology. In this regard, essential roles of Foxp3^+^ Treg cells in sustaining homeostasis of diverse tissues are now emerging, although much knowledge about these non-canonical tissue-specific functions is still to be obtained (95). In the intestine, Foxp3^+^ Treg cells are involved in preserving the function and homeostasis of intestinal epithelial cells (IEC) (**Figure 2**). Positioned as a physical barrier between the intestinal lumen and the immune cells in the lamina propria, IEC spatially segregate host and microbiota (96). At the same time IEC facilitate the crosstalk between microbes and host cells by sensing and responding to immune as well as microbial stimuli (96).

Foxp3^+^ Treg cells promote IEC homeostasis by supporting epithelial stem-cell renewal (97). In an *in vitro* organoid system, addition of Treg cells or their major effector cytokine IL-10 supported stem-cell renewal (97). *In vivo*, depletion of Treg cells decreased intestinal stem cell proportions while higher differentiation rates of IEC were observed (97). Interestingly, IL-10 was also shown to maintain IEC function by regulating their fucosylation and by protecting IEC from endoplasmic reticulum stress as well as from Fas-mediated apoptosis (98–101), although the precise cellular source of IL-10 was not elucidated in these studies. In addition to direct effects on IEC, intestinal Foxp3^+^ Treg cells shape IEC function also indirectly by controlling e.g. the abundance of IL-22-producing Th17 cells in the gut (31–34, 36, 90). IEC constitutively express the IL-22 receptor, and IL-22 signalling in IEC is critical for maintaining the integrity of the mucosal barrier (102).

Conversely to the effect of Treg cells on IEC, signals derived from IEC also influence the function and abundancy of Foxp3^+^ Treg cells in the lamina propria, thereby establishing a reciprocal regulatory circuit (**Figure 2**). For instance, intestinal ST2^+^ GATA3^+^ tTreg cell function is boosted by the release of IL-33 upon IEC damage (see section above) (66, 67). Another example comes from a study analysing the effects of IEC apoptosis on intestinal Treg cell homeostasis, in which apoptotic IEC reduced the abundancy of gut-resident Foxp3^+^ Treg cells, thus lowering the threshold for inflammatory immune responses (103). Even expansion of intestinal Treg cells induced by direct antigen-driven interaction with IEC has been suggested (104, 105), although the role of IEC antigen presentation in shaping intestinal immunity has not been thoroughly explored so far. Recently, another unconventional interaction of Treg cells with IEC has been identified. Upon migration to the epithelium, intestinal pTreg cells were shown to downregulate Foxp3 and convert to intraepithelial (IEL) CD4^+^ T cells in order to control intestinal inflammation (106). These findings reveal an unprecedented phenotypic and functional adaptability of intestinal Treg cells. Moreover, they demonstrate a dominant role of the IEC microenvironment in controlling Treg cell lineage stability and plasticity, highlighting the close interdependence between Treg cells and IEC (106).

## Concluding Remarks

It is now well established that intestinal Foxp3^+^ Treg cells are critical for the tolerance to commensal microbes, the induction of oral tolerance and for host defense against enteric pathogens, thereby installing gut immune homeostasis. Beyond these classical immune-related functions, novel roles of Treg cells in gut organismal homeostasis are emerging, unrevealing a greater functional and phenotypic diversity of the intestinal Treg cell compartment than was previously recognized. Given these non-canonical functions in tissue maintenance, regeneration and repair, intestinal Treg cells can be considered not only as mediators of immunological tolerance but also of disease tolerance, a concept, which encompasses multiple mechanisms that help decrease host susceptibility to tissue damage during pro-inflammatory immune responses (107, 108).

Clearly, we are just beginning to understand the impact of Treg cells on gut physiology and much remains to be uncovered about the relationship and crosstalk between Treg cells and distinct intestinal tissue cells, such as endothelial, epithelial, stromal or neuronal populations. From a translational point of view, impaired intestinal Treg cell functionality is associated with chronic inflammatory diseases, such as inflammatory bowel disease and food allergy. Thus, further explorations into the characteristics, dependencies and targets of different intestinal Treg cell subsets will undoubtedly help to develop more targeted manipulation strategies, aiming at a selective enhancement or inhibition of Treg cell function in a context- and tissue-specific manner.

## Author Contributions

CC and CN wrote the manuscript. CC generated the figures. CN provided the overall design and guidance for this Review. All authors contributed to the article and approved the submitted version.

## Funding

We acknowledge support from the German Research Foundation (DFG) and the Open Access Publication Fund of Charité – Universitätsmedizin Berlin.

## Conflict of Interest

The authors declare that the research was conducted in the absence of any commercial or financial relationships that could be construed as a potential conflict of interest.
